# Case Report: The role of bone scans in detecting Ribbing disease

**DOI:** 10.3389/fnume.2025.1527159

**Published:** 2025-03-12

**Authors:** Abel Dambrain, Clément Bouron, Franck Lacoeuille

**Affiliations:** ^1^ Department of Nuclear Medicine, University Hospital Center, Angers, France; ^2^ CRCI2NA—Inserm UMR1307/CNRS UMR 6075, University of Angers, Angers, France

**Keywords:** Ribbing disease, osteocondensation, bone scan, osteoarticular, dysplasia, scintigraphy

## Abstract

In this case, we report the usefulness of bone scintigraphy in evaluating osteoarticular pain when the diagnosis is unclear after standard morphological imaging. A 24-year-old male patient exhibited mild left tibial pain that had been intensifying over a period of 2 years. The initial radiological evaluation suggested a diagnosis of pediatric tibial bone marrow osteosclerosis associated with periostitis, based on standard radiographs and MRI. However, a complementary bone scan was required for confirmation and showed moderate hyperemia and severe hyperfixation of the tibial lesion along with similar lesions on the left femur, both humeri, and the right ulna. These new findings led to a diagnosis of Ribbing disease, a rare sclerosing bone dysplasia.

## Introduction

Ribbing disease may be responsible for chronic osteoarticular pain that can lead to significant disability. There are no specific tests to confirm the diagnosis, making imaging essential for excluding other differential diagnoses (1, 2).

Typically, the lesions are asymmetrical, affecting only the long bones (most frequently the tibia, followed by the femur and humerus), sparing the epiphyses. CT scans typically show a medullary and periosteal thickening, and MRI shows medullary edema (2, 3). As in our case, these characteristics could be compatible with several other pathologies, such as periostitis and sclerosing bone dysplasia. A bone scan provides osteoblastic information of the whole body, which may help to refine the diagnosis. In the case of Ribbing disease, hyperemia is associated with irregular hyperfixation (1, 4) of lesions due to the increase in osteoblastic activity. This case highlights the role of bone scintigraphy as part of the diagnostic approach, facilitating the identification of Ribbing disease and excluding other conditions, such as periostitis or osteosclerosis.

## Case

A 24-year-old male patient presented with diurnal pain for 2 years, often associated with exercise, but not disabling. He had no personal medical history but had an uncle with chronic diffuse pain that was not investigated. The clinical examination only revealed an anterior tibial swelling and blood tests were normal.

**Figure 1:**
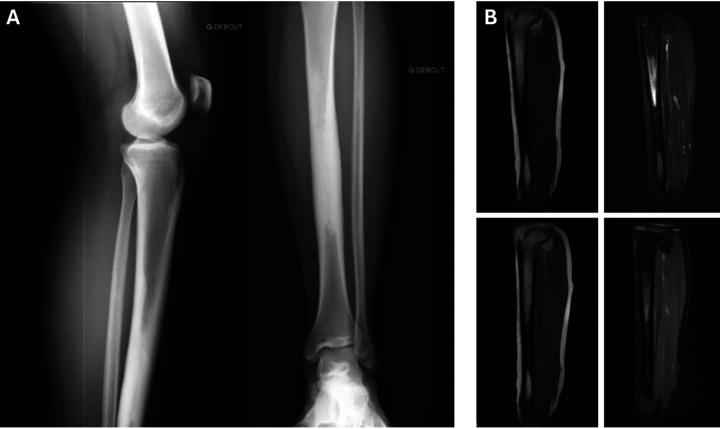
X-ray **(A)** and MRI **(B)** of the left tibia showing intramedullary osteosclerosis associated with peripheral edema.

X-ray imaging (Figure 1A) revealed endosteal medio-diaphyseal osteocondensation of the left tibia extending over 12 cm, sparing the metaphysis and epiphyses. MRI (Figure 1B) showed a circumferential sclerotic lesion hypointense on T1 and T2 weighted images with medullary canal narrowing, associated with bone marrow and periosteal edema. There were no abnormalities of the soft tissue nor any signs of malignant periosteal reaction. The radiologists concluded that there was intramedullary osteosclerosis of the tibia associated with medial tibial stress syndrome (periostitis stage).

**Figure 2:**
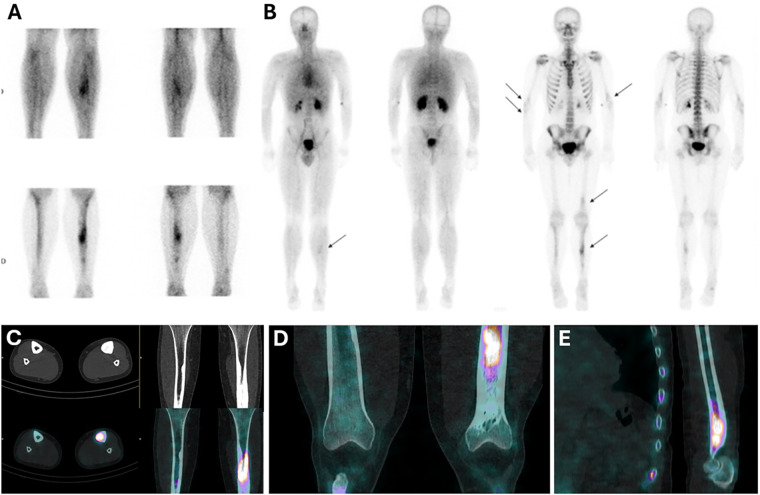
Bone scintigraphy showing hyperemia **(A)** and hyperfixation in the tibias, left femur, and humeri **(B)**, associated with intramedullary osteosclerosis on CT scans **(C–E)**.

A [^99m^Tc]Tc-HDP (hydroxymethylene diphosphonate) single-photon emission computed tomography (SPECT)-CT scan (Figure 2) was requested to specify the diagnosis. It showed moderate hyperemia associated with severe and heterogeneous hyperfixation of the left tibial lesion (Figure 2A). In addition, the whole-body scan (Figure 2B) revealed moderate hyperfixation in other parts of the skeleton, including the lower third of the left femur, the distal extremities of both humeri, and the upper part of the right ulna. CT images showed a similar appearance to the intramedullary condensing tibial lesion (Figure 2C) at the other sites (Figures 2D,E) with an important osteoblastic reaction.

The patient did not undergo a biopsy, but the biological and radiological data supported the diagnosis of Ribbing disease as a diagnosis of exclusion. A radiological follow-up performed 1 year later showed stable lesions, and the patient experienced no worsening of symptoms during this period despite the absence of specific treatment, consistent with Ribbing disease.

## Discussion

Ribbing disease is a sclerosing diaphyseal dysplasia, first recognized by Ribbing in 1949 (5), primarily affecting young patients. Its origin is related to an autosomal recessive mutation of the *TGF-β1* gene, which is responsible for increased differentiation and proliferation due to the activation of growth factors such as TNF-α (tumor necrosis factor-α), INF-γ (interferon-γ) and TGF-b (transforming growth factor bêta). Thus, a family history is present in up to 50% of cases (1). It may be asymptomatic, or the only clinical abnormality may be localized pain caused by local swelling. Blood tests should be normal and exclude bone remodeling, vitamin deficiency, or infection. Histology is non-specific but contributes to excluding osteomyelitis or neoplasia. Biopsy should reveal an increase in osteoblastic activity associated with fibrosis and Haversian system disorders (1). Bone scintigraphy contributes to the diagnosis by identifying fixation patterns that exclude some infectious, inflammatory, or tumor processes. It also allows for an efficient topographic analysis of lesions across the whole body, which helps to guide biopsy procedures for histological analysis. In our case, the initial diagnosis was periostitis associated with medullary osteosclerosis due to the shared characteristics of these three diseases and their prevalence in the general population. Ribbing disease can be distinguished from periostitis by the presence of intense non-linear uptake, along with the absence of fracture on all imaging modalities (6). The clinical context also differs, as periostitis typically occurs following a recent increase in physical activity. In comparison with osteosclerosis, Ribbing disease usually shows medullary edema adjacent to areas of osteosclerosis on MRI and intense hyperemia on bone scans (7, 8). These characteristics are summarized in Table 1. Another differential diagnosis is Camurati–Engelmann disease, which is a progressive sclerosing diaphyseal dysplasia with autosomal dominant inheritance, described by Cockayne in 1920 (9) and caused by a TGF-β1 mutation. It is characterized by the same lesion as Ribbing disease but is symmetrical and associated with a physical disability such as a Marfanoid profile, myopathic symptoms, and nerve damage (10).

**Table 1:** Main differences between Ribbing disease, periostitis, and intramedullary osteosclerosis according to different imaging modalities (CT, MRI, and bone scintigraphy) (1–3, 6–8).

Imaging modality	Ribbing	Periostitis	Medullar osteosclerosis
X-ray and CT scan	Intramedullary density Periosteal thickening Endosteal thickening	Normal Periosteal thickening Fracture	Soft tissue swelling Periosteal thickening Endosteal thickening
MRI	Decreased signal on T1 and T2 weighted images Cortical thickening Peripheral endosteal bone marrow edema Adjacent soft-tissue edema	Endosteal bone marrow edema Cortical edema (increased signal on T2) Areas of intracortical signal changes Fracture	T1: decreased signal intensity T2: variable Gadolinium: low signal intensity at the bone site
Bone scan	Intense irregular uptake Moderate to intense hyperemia	Moderate uptake, heterogeneous and linear Variable periosteal hyperemia Fracture: focal hyperemia	Moderate uptake Slightly increased vascularity

There are no recommendations for the therapeutic management of Ribbing disease. Available treatments include analgesics, non-steroidal anti-inflammatory drugs (NSAIDs), corticosteroids, and bisphosphonates, but their efficacy is highly debated. As a last resort, intramedullary reaming may be considered to reduce intraosseous pressure and edema caused by osteosclerosis, with notable effectiveness (2).

## Conclusion

Our case highlights the importance of dual-phase bone scintigraphy in patients with the common clinical features of chronic osteoarticular pain and atypical lesions on CT and MRI, in whom Ribbing disease should be considered despite its rarity.

## Data Availability

The raw data supporting the conclusions of this article will be made available by the authors, without undue reservation.
